# Effects of Adult Age and Functioning of the Locus Coeruleus Norepinephrinergic System on Reward-Based Learning

**DOI:** 10.1523/JNEUROSCI.2006-22.2023

**Published:** 2023-08-30

**Authors:** Hsiang-Yu Chen, Michael Marxen, Martin J. Dahl, Franka Glöckner

**Affiliations:** ^1^Lifespan Developmental Neuroscience, Faculty of Psychology, Technische Universität Dresden, 01062 Dresden, Germany; ^2^Methods of Psychology and Cognitive Modeling, Faculty of Psychology, Technische Universität Dresden, 01062 Dresden, Germany; ^3^Department of Psychiatry and Psychotherapy, Technische Universität Dresden, 01062 Dresden, Germany; ^4^Center for Lifespan Psychology, Max Planck Institute for Human Development, 14195 Berlin, Germany; ^5^Davis School of Gerontology, University of Southern California, Los Angeles, Los Angeles, California 90089

**Keywords:** aging, decision-making, locus coeruleus, norepinephrine, pupil dilation, reinforcement learning

## Abstract

Age-related impairments in value representations and updating during decision-making and reward-based learning are often related to age-related attenuation in the catecholamine system such as dopamine (DA) and norepinephrine (NE). However, it is unclear to what extent age-related declines in NE functioning in humans affect reward-based decision-making. We conducted a probabilistic decision-making task and applied a Q-learning model to investigate participants’ anticipatory values and value sensitivities. Task-related pupil dilations and locus coeruleus (LC) magnetic resonance imaging (MRI) contrast, which served as a potential window of the LC-NE functions, were assessed in younger and older adults. Results showed that in both choice and feedback phases, younger adults’ (*N* = 42, 22 males) pupil dilations negatively correlated with anticipatory values, indicating uncertainty about outcome probabilities. Uncertainty-evoked pupil dilations in older adults (*N* = 41, 27 males) were smaller, indicating age-related impairments in value estimation and updating. In both age groups, participants who showed a larger uncertainty-evoked pupil dilation exhibited a higher value sensitivity as reflected in the β parameter of the reinforcement Q-learning model. Furthermore, older adults (*N* = 34, 29 males) showed a lower LC-MRI contrast than younger adults (*N* = 25, 15 males). The LC-MRI contrast positively correlated with value sensitivity only in older but not in younger adults. These findings suggest that task-related pupillary responses can reflect age-related deficits in value estimation and updating during reward-based decision-making. Our evidence with the LC-MRI contrast further showed the age-related decline of the LC structure in modulating value representations during reward-based learning.

**SIGNIFICANCE STATEMENT** Age-related impairments in value representation and updating during reward-based learning are associated with declines in the catecholamine modulation with age. However, it is unclear how age-related declines in the LC-NE system may affect reward-based learning. Here, we show that compared with younger adults, older adults exhibited reduced uncertainty-induced pupil dilations, suggesting age-related deficits in value estimation and updating. Older adults showed a lower structural MRI of the LC contrast than younger adults, indicating age-related degeneration of the LC structure. The association between the LC-MRI contrast and value sensitivity was only observed in older adults. Our findings may demonstrate a pioneering model to unravel the role of the LC-NE system in reward-based learning in aging.

## Introduction

Learning is one of the major mechanisms needed to acquire the value of information in a new environment (Yu and Dayan, 2005; Behrens et al., 2007; Rushworth and Behrens, 2008). However, if the environment contains uncertain information that cannot be easily observed, individuals apply trial and error strategies to update their value representations according to previous outcomes until they collect knowledge about the hidden information in the environment. This uncertainty in the environment exposes individuals to the differences between predicted and received rewards, and their mismatch, also called reward prediction error (RPE), may drive learners to update their predicted values to maximize their rewards. Value representations and RPE during reward-based learning are shown to relate to catecholaminergic neuromodulation, which includes dopamine (DA) (Pessiglione et al., 2006; Schlagenhauf et al., 2013; Schott et al., 2008) and norepinephrine (NE) (Jahn et al., 2020; Luksys et al., 2009) in the frontostriatal network.

In the course of healthy aging, empirical evidence has shown impairments of value representations and updating during decision-making and reward-based learning in old age (Eppinger and Bruckner, 2015; Eppinger and Kray, 2011; Frank and Kong, 2008; Hämmerer et al., 2011; Simon et al., 2010; Sojitra et al., 2018). The changes in value representations and deficits in value updating observed in older adults are presumably related to the age-related attenuation of the DA and NE systems in the frontostriatal network (Eppinger and Bruckner, 2015; Samanez-Larkin and Knutson, 2015). Indeed, neuroimaging studies reported that compared with younger adults, older adults showed reduced DA receptor bindings in the nucleus accumbens during reward-based decision-making (Chowdhury et al., 2013; de Boer et al., 2017; Dreher et al., 2008), suggesting age-related impairments in value representations. However, it is not clear to what extent the age-related decline in NE functioning in humans may affect decision-making and reward-based learning.

In the present study, we conducted a probabilistic decision-making task and applied a Q-learning model (Sutton and Barto, 2018; Van Slooten et al., 2018) in younger and older adults to investigate how age may affect learning tendency and value sensitivity during reward-based learning and its association with LC-NE functioning. We also assessed individual participants’ pupil dilations during the task and the T1-contrast structural magnetic resonance imaging (MRI) of the LC (hereafter referred to as LC-MRI contrast) to serve as potential indicators of LC-NE functioning. Although the association between pupillary responses and tonic LC activity is still disputed (Breton-Provencher and Sur, 2019; Privitera et al., 2020; cf. Megemont et al., 2022), some studies have shown that task-related pupil dilations positively correlated with the phasic activity of LC neurons in animals (Joshi et al., 2016; Reimer et al., 2016; Usher et al., 1999; Megemont et al., 2022) and humans (de Gee et al., 2014; Murphy et al., 2014). Despite these caveats, task-related pupil dilation serves as an integrated and robust readout of the psychophysiological toolbox (Strauch et al., 2022). Specifically, Van Slooten et al. (2018) demonstrated that when performing a probabilistic decision-making task that involves reward-based learning, pupil dilations can reflect value beliefs of the choice options and RPEs in younger adults. Therefore, we examined to what extent task-related pupil dilations may reflect the mechanism of aging in changing value representations and updating during reward-based decision-making. Furthermore, we assessed the LC-MRI contrast as an indicator of LC-NE functioning. Recent advancements in MRI approaches allow for the visualization and quantification of the LC structure. The LC-MRI contrast is evidenced to track the degeneration of the LC in Alzheimer’s disease (Dahl et al., 2022; Jacobs et al., 2021b) and showed positive associations with cognitive functions such as memory (Dahl et al., 2019; Hämmerer et al., 2018) or attentional shifting ability (Clewett et al., 2016) in cognitively normal older adults. The findings suggest that the LC-MRI contrast may be a window to indicate age-related LC-NE functioning during reward-based learning.

In light of previous findings (Frank et al., 2007; Hämmerer et al., 2011), we hypothesized that relative to younger adults, older adults would show worse choice performance in selecting higher rewarded options and exhibit lower value sensitivity to outcome probabilities. We tested age differences in task-related pupil dilations to understand the age-related changes in value estimation and updating. Finally, we expected that individuals’ value sensitivities to reward probabilities would positively correlate with individual differences in NE functioning as reflected in task-related pupil dilations and LC-MRI contrasts.

## Materials and Methods

### Participants

Forty-six younger adults (25 males; age range, 20–36 years; mean ± SD, 27.37 ± 5.16 years) and 49 older adults (34 males; age range, 66–81 years; mean ± SD, 72.67 ± 4.46 years) participated in the study. All participants had normal or corrected-to-normal vision, and none of them reported a history of psychiatric or neurologic disorders. Pupillometry and neuroimaging data were collected at two separate time points. Because alcoholic and caffeinated substances affect physiological variables such as pupil size (Holmqvist et al., 2011), participants were asked to refrain from consuming alcohol for 72 h before the pupillometry experiment day and from drinking any caffeinated drinks (e.g., coffee or tea) on the day of the pupillometry experiment. Experimental assessments were conducted between 9:00 A.M. and 5:00 P.M. to minimize the potential effects of the circadian rhythm on pupil dilations (Markwell et al., 2010). Data from four younger and eight older adults whose eye-tracking data quality was poor (>50% of the data needed to be removed from the analyses of pupil size) were included in the analyses of choice behaviors but excluded from the pupillometry analyses. Furthermore, 25 (of 46) younger (15 males) and 34 (of 49) older adults (29 males) participated in an additional MRI session to obtain their structural whole-brain and brainstem scans for assessing the LC-MRI contrast. Written informed consent in accordance with the revised Declaration of Helsinki (2008) was obtained from all participants before the experiment. The study was approved by the ethics committee of Technische Universität Dresden (EK 511112015). Each subject received 15 euros as compensation for participation in the pupillometry experiment (duration, 2 h) plus a monetary bonus, which was computed from the total points that the participant obtained in the probabilistic decision-making task and converted into Euro cents. If subjects participated in the additional MRI session (duration, 30 min), an additional 5 euros was added. Table 1 depicts the demographic and sample characteristics of younger and older participants. Specifically, younger adults showed better performance than older adults in processing speed (measured by the Identical Picture test; Lindenberger and Baltes (1994) but showed worse performance than older adults in verbal knowledge (measured by the Spot-A-Word test; Lindenberger and Baltes, 1994) as previously reported.

**Table 1:** Demographic characteristics, basic cognitive abilities, and performance by age group

	Younger Adults	Older Adults	Statistics
N	46	49	
Age (years)	27.37 (5.16)	72.67 (4.46)	
Gender (%male)	54	69	*χ^2^*_(1)_ = 2.56 (*p* = 0.11)
Years of Education	16.97 (3.25)	15.30 (4.24)	*t*_(85.19)_ = 2.31 (*p* = 0.02)
Verbal Knowledge: SaW	22.80 (3.16)	26.22 (3.72)	*t*_(91.46)_ = −4.82 (*p* < 0.001)
Processing Speed: IPT	31.69 (4.63)	20.78 (4.33)	*t*_(89.93)_ = 10.67 (*p* < 0.001)

Note. Age group difference for gender ratio was examined using a Chi-square test. Age group differences for other variables were examined using two independent sample t-tests with Welch–Satterthwaite approximation on the degree of freedom. Abbreviations: IPT = the number of correct responses in the Identical Pictures task (Lindenberger and Baltes, 1994); SaW = the number of correct responses in the Spot-a-Word task (Lindenberger and Baltes, 1994). Note: Standard deviations in parentheses.

### Apparatus and stimuli setup

The experimental task was programmed in MATLAB R2016b (version 9.1, MathWorks) with Psychtoolbox software (Brainard, 1997). Stimuli were displayed on a 23-inch monitor and viewed from a distance of 60 cm. The illumination of the background environment was fixed at 300 lux to control for potential luminance-related effects on pupil size. Furthermore, the head position of the participants was stabilized using a chin and forehead rest.

### Probabilistic decision-making task and procedure

**Figure 1.**
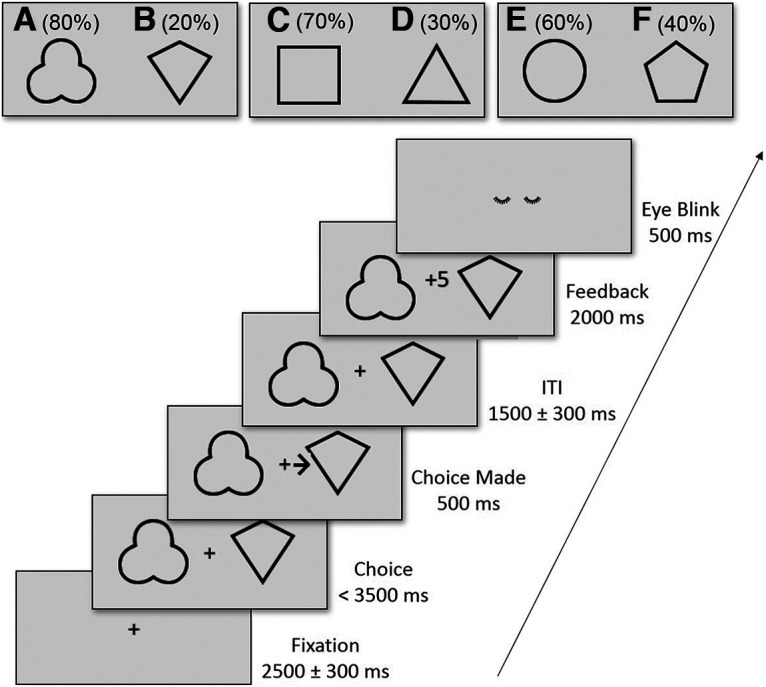
Schematic diagram of the probabilistic decision-making task. In the task participants were presented with three choice pairs and were asked to maximize reward points by learning from the previous probabilistic feedback, indicating +5 or −5 reward points after a choice was made. Reward probabilities are indicated in the figure. Choosing option A, for example, resulted in a reward probability of 80%, whereas choosing option B resulted in a reward probability of only 20%.

The probabilistic decision-making task was adapted from Van Slooten et al. (2018) with the following reward probability pairs: 80/20% for AB, 70/30% for CD, and 60/40% for EF choice options. Figure 1 depicts the task design and procedure. Each pair consists of two black geometric shapes with a gray background. The total of six geometric shapes was randomly coupled to the three reward probability mappings (AB, CD, EF), and the order of the reward probabilities in a pair was counterbalanced (e.g., 80/20% for AB or 80/20% for BA) across participants. Three choice pairs were presented in a pseudorandomized order across the trials so that no more than three consecutive trials showed the same choice pair. Each trial started with a fixation cross at the center of the screen with a mean duration of 2500 ms (SD = 300 ms), followed by a choice phase. In the choice phase, two geometric shapes appeared at the horizontal meridian left and right from the central fixation cross. Participants were asked to make a choice between the two options as quickly as possibly by pressing the M or Y key on a German keyboard using the right and left index fingers, respectively. After a choice was made or until a response deadline of 3500 ms was met, a small black arrow was instantly shown for 500 ms pointing to the choice option the participant selected, followed by a random interval with a mean duration of 1500 ms (SD = 300 ms). After the random interval, the feedback phase began with the outcome shown at the middle between the choice options indicating a gain or loss of five points for 2000 ms, followed by an eyeblink symbol for 500 ms, instructing the participants to quickly blink at the end of each trial. The eyeblink event aimed to reduce the probability of blinks during trials that might cause pupillometry data loss during the choice or feedback phase. Each run consisted of 60 trials, with 20 repetitions of each choice pair. Altogether, there were six runs and a total of 360 trials.

### Reinforcement learning (Q-learning) model

Choices in valid trials (with reaction times >150 ms and <3500 ms) were fit with a Q-learning model. During Q-learning (also called model-free learning), individuals did not know about the probability of obtaining reward points in each choice option. However, to maximize the reward points, they had to update the value beliefs (Q-values) of the chosen options by learning from the feedback on previous choices. Therefore, the model estimates the value belief of each choice option based on a series of the participants’ choices and received outcomes. All Q-values were initially set to 0.5. For each choice, the Q-value of the chosen option is updated by learning from the feedback that yields unexpected positive or negative RPE (δ; Eq. 1). Thus, the Q-value of the option i in the next trial *t + 1* can be updated by the outcome r of the current choice and be formulated as follows:

$$\delta (t)=r_{i}(t)-Q_{i}(t)$$

$$Q_{i}(t+1)=Q_{i}(t)+\{\begin{array}{c} \alpha_{\mathrm{gain}}\times \delta (t)\;if\;r=1\\ \alpha_{\mathrm{loss}}\times \delta (t)\;if\;r=0 \end{array},$$

where α_gain_ and α_loss_ indicate positive and negative learning rates, respectively, to determine the extent to which the Q-value of the chosen option is updated by the RPE, δ. Because previous neuroimaging studies have shown that different subgroups of striatal neurons are separately involved in positive or negative feedback learning (Frank, 2005; Shen et al., 2008), we modeled two separate learning rates. Given the Q-values of the two presented stimuli in a pair, the choice on the next trial was described by a softmax function, which can be formulated as follows:

$$P_{A}(t)=\frac{\exp(\beta \times Q_{A}(t))}{\exp(\beta \times Q_{A}(t))+\exp(\beta \times Q_{B}(t))},$$

where β is an inverse temperature parameter (also known as exploitation-exploration tendency or value sensitivity) to indicate how deterministic the choices are. Higher β values indicate greater sensitivity to reward probabilities and higher exploitations to the higher rewarded options.

Furthermore, the Q-learning model was applied using a hierarchical Bayesian approach that was adapted from Van Slooten et al. (2018) to approximate the value of each parameter. Specifically, with the hierarchical Bayesian fitting approach, individual parameter estimates were drawn from group-level parameter distributions separately for each age group to constrain the range of individual parameter estimates. We assumed participants’ performance in each age group came from a normally distributed population, thus a normally distributed prior was assigned for each parameter. This approach allows for the simultaneous estimation of the group- and individual-level parameters to enhance statistical strength as well as take individual differences into account (Lee, 2011). Finally, the mean of the posterior distribution for each parameter was used for further statistical comparisons.

To assess whether the hierarchical Bayesian Q-learning model is capable of reliably identifying the effects of adult age, we ran a parameter recovery analysis. First, the mean parameter values (true parameters) of each participant were used to fit a generative version of the model to simulate the behavioral datasets. Next, we used our model-fitting procedure to fit the simulated behavioral datasets to obtain the estimated parameters. As the mean of the posterior distribution for each parameter at the individual level was submitted to test age differences and further examine the relationships between β parameter values of the Q-learning model and NE-associated psychophysiological indicators such as task-related pupil dilations and LC-MRI contrast (see below, Statistical analyses), the correlation analyses between the true and estimated parameters at the individual level were applied. We found substantial correlations between true and estimated parameters in both age groups (all *r* values *>* 0.9, Table 2). The findings indicate that our model can represent meaningful parameter estimates for age and individual differences in value sensitivity and learning types during the probabilistic decision-making task.

**Table 2:** Correlation coefficients between the true and estimated parameters of the hierarchical Bayesian Q-learning model at the individual level

Parameter age group	β	α_gain_	α_loss_
Younger adults	0.91	0.89	0.96
Older adults	0.94	0.90	0.92

### Acquisition and preprocessing analysis of pupillometry data

Participants’ pupil size was continuously recorded during the task using a Tobii TX300 eye tracker (Tobii Technologies) with a sampling rate of 300 Hz. A five-dot eye-tracking calibration was conducted at the beginning of each task run. During preprocessing, pupillometry data were segmented into two time windows. One was segmented 200 ms before and 3000 ms after the paired-choice options were shown, which was associated with value comparison in the decision phase. The other one was segmented 200 ms before and 3000 ms after feedback onset related to value updating. A customized Python script detected eyeblinks in the segmented data. The periods of missing data because of blink artifacts were segmented in a time window of 100 ms before and 100 ms after the blink and replaced by a linear interpolation. As such, the interpolation was only applied to periods where data loss durations were shorter than 750 ms (Chen et al., 2021; Dix and Van der Meer, 2015). Trials with excessively noisy or missing data in which the blink artifacts sustained over 750 ms were excluded within subject (removed choice events, mean ± SD = 0.03 ± 0.17 in younger and 0.08 ± 0.27 in older adults; removed feedback events, mean ± SD = 0.09 ± 0.29 in younger and 0.14 ± 0.35 in older adults). Pupillometry data were then baseline corrected with regard to the first 200 ms of each segmented time window, and standardized *z* scores were calculated within participant to allow comparing pupil dilations associated with value beliefs or RPE independent of individual differences in mean and variance of pupil size (Hämmerer et al., 2017, 2019; Van Slooten et al., 2018).

### Acquisition of structural MRI data and LC-MRI contrast assessment

Structural MRI scans were acquired on a 3T Tim Trio whole-body scanner (Siemens) with a standard 32-channel head coil used for signal reception. A high-resolution T1-weighted anatomic image was acquired using a magnetization prepared rapid gradient echo sequence for each participant. The parameters for the T1-weighted MPRAGE were as follows: voxel size = 0.85 × 0.85 × 0.85 mm, 240 slices, TR = 2400 ms, TE = 2.19 ms, flip angle = 8°, FOV = 272 × 272 mm, bandwidth = 210 Hz/pixel, acquisition matrix = 320 × 320. Furthermore, brainstem scans were acquired with a 3D T1-weighted multiecho fast low-angle shot (FLASH) acquisition (Hämmerer et al., 2018). The voxel size was 0.4 × 0.4 × 3 mm adjusted to the geometry of the LC area with 3-mm-thick slices perpendicular to the long axis of the LC. Protocol parameters were as follows: TE1 = 5.33 ms, TE2 = 11.62 ms, flip angle = 15°, FOV = 128 × 128 mm, bandwidth = 130 Hz/pixel, acquisition matrix = 320 × 320. Each brainstem scan consisted of 30 axial slices with a gap of 20% between slices. To improve the signal-to-noise ratio (SNR), this acquisition was repeated three or six times for each participant, but only the first three scan sessions within each participant were used for further analysis.

**Figure 2.**
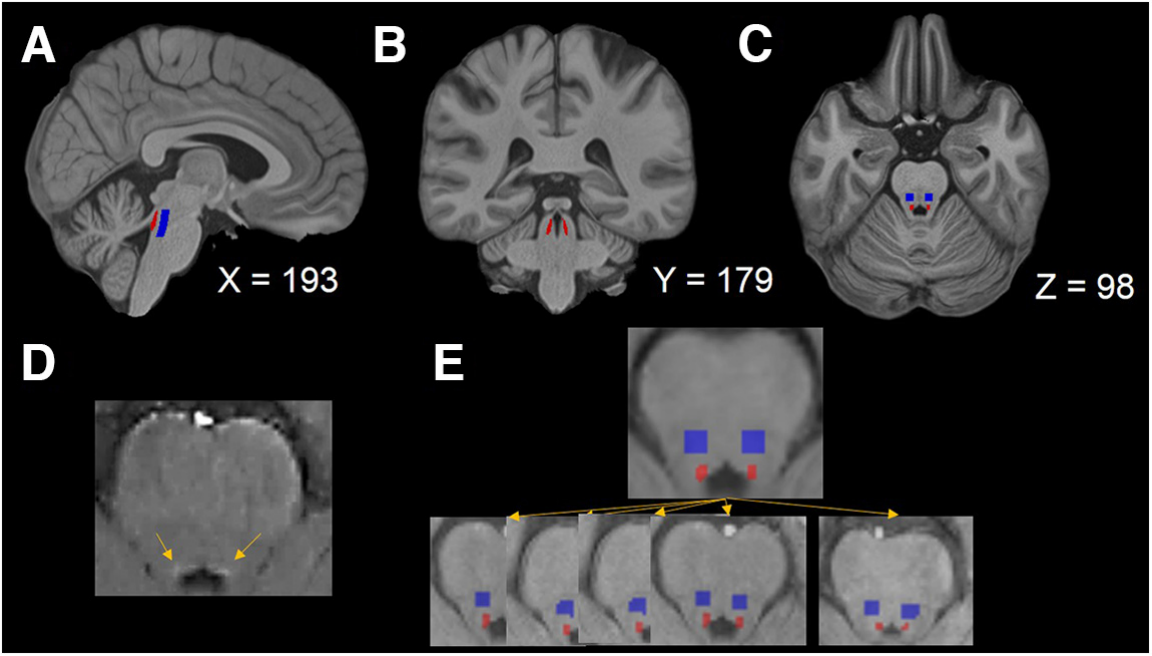
LC and pontine reference regions of interest. ***A–C***, Sagittal, coronal, and axial views of the MNI 0.5 mm linear template with the LC (red; Dahl et al., 2022) and the lateralized pontine reference (blue) mask. ***D***, Visualization of the LC in one participant’s FLASH brainstem image (yellow arrows indicating the LC). ***E***, The LC and pontine reference masks in the MNI 0.5 mm linear space (top) were wrapped back to individual participants’ FLASH images (bottom) that were moved to their native whole-brain space.

Signal intensities in the LC area were extracted using the binarized LC mask published by Dahl et al. (2022), which was in the MNI 0.5 mm linear space, on all participants’ FLASH brainstem images (Fig. 2*A–C*). Moreover, a pontine reference mask with 4 × 4 mm was created in which the dorsal boundary of the pontine reference region was determined by moving 12 voxels (6 mm in the MNI 0.5 mm linear space) from the dorsal end of the LC region toward the pons. Individual participants’ FLASH brainstem images (Fig. 2*D*) were aligned to their native whole-brain image (Step 1). All participants’ whole brain images were coregistered to the MNI 0.5 mm linear space where the LC and pontine reference masks were to obtain the transformation matrices (Step 2). For the coregistrations in Steps 1 and 2, an antsRegistration function in Advanced Normalization Tools (version 2.1; Avants et al., 2009) was performed with a linear registration (rigid, then affine) and followed by nonlinear registrations (symmetric normalization). We further applied the transformation matrices obtained from Step 2 to move the LC and pontine reference masks in the MNI 0.5 mm linear space using the nearestNeighbor interpolation back to individual participants’ FLASH brainstem images (Betts et al., 2017) that were moved to their whole-brain space to extract signal intensities (Fig. 2*E*).

To reduce signal noises between participants as well as allow normalization and between-participant comparisons, the signal intensity in the LC masked region was compared with the signal intensity in a pontine reference area according to the previous literature (Bachman et al., 2021; Betts et al., 2017; Clewett et al., 2016; Dahl et al., 2019). The pontine reference area was selected because it is adjacent to the LC but does not have NE neurons to generate neuromelanin that shortens T1 effects on the brainstem images (Liu et al., 2017). To obtain the LC-MRI contrast ratio, we applied the binarized LC and pontine reference masks to the individual’s brainstem scans (that were moved to their whole-brain native space) to extract the maximal signal intensity values in the masked regions across the rostrocaudal extent of the LC for each session (three FLASH brainstem scan sessions in total). The LC contrast ratio was calculated as follows (Bachman et al., 2021; Betts et al., 2017; Dahl et al., 2019, 2022):

$$LC_{ratio}=\frac{\left(max(S_{LC})-max(S_{Ref})\right)}{max(S_{Ref})},$$

where *max*(*S_LC_*) denotes the maximal signal intensity of the left or right LC and *max*(*S_Ref_*) indicates the maximal signal intensity of the pontine reference region in the corresponding slice where the maximal signal intensity of the left or right LC was.

### Statistical analysis

Statistical comparisons for the choice performance and parameter estimates of the Q-learning model were conducted using R software (version 4.0.2). The distributions of the data or those of ANOVA residuals were examined for normality using the Shapiro–Wilk test with the shapiro.test function in R. If the residuals of ANOVAs were not normally distributed, an aligned rank transform ANOVA, which is a nonparametric approach to factorial ANOVA (Wobbrock et al., 2011), was conducted for the main and interaction effects using the art function in the ARTool package. *Post hoc* tests were conducted to evaluate the contrasts and were Bonferroni corrected at a significance level of *p* < 0.05 using the emmeans package in R. Effect sizes (partial *η*^2^ or *d*) were calculated using the effectsize package in R. For the pupillometry data, nonparametric cluster-based permutation *t* tests were applied using the MNE (version 0.20.7) package in Python, and the correlation analyses were conducted using the scipy.stats package (version 1.5.0) in Python (version 3.6.10).

#### Age differences in choice performance and parameters of the Q-learning model

Accuracy of selecting the higher rewarded options was computed as the number of trials in which the participant correctly selected the higher reward option divided by the number of valid trials in each of the three choice pairs. For age differences in the accuracy of selecting higher rewarded options at the behavioral level, an aligned rank transform (nonparametric) ANOVA with age group as the between-subject factor (younger/older adults) and choice pair (80–20/70–30/60–40) and experimental run (1–6) as within-subject factors was applied. Adult age differences in value sensitivity (β parameter values from the Q-learning model) were examined using the Mann–Whitney *U* test. Moreover, the learning rates of gain and loss were submitted to a 2 (age group) × 2 (learning rate) aligned rank transform ANOVA. *Post hoc* tests were conducted with Bonferroni correction for multiple comparisons at a significance level of *p <* 0.05.

#### Age differences in the pupillometry data

Preprocessed pupillometry data were submitted to general linear models (GLMs) to investigate how value beliefs were associated with task-related pupil dilations in the choice and feedback phases. Continuous data points of pupil dilations in the time window within 3 s after choice pair onset were selected as dependent variables, and the trial-by-trial Q-value differences between the paired-choice options were converted into standardized *z* scores as independent variables within subject (Eq. 5). For the time window within 3 s after feedback onset, the pupillometry data were submitted as dependent variables, and the Q-values of the chosen options were converted into *z* scores and used as the independent variable (Eq. 6). In addition, the trial-by-trial RPE estimates were also converted into *z* scores and submitted as another independent variable (Eq. 6). The regression models in the choice and feedback phases were applied to individual participants’ data and formulated as follows:

$$PD_{t}=\beta_{0}+\beta_{1}\Delta Q+\varepsilon$$

$$PD_{t}=\beta_{0}+\beta_{1}Q_{chosen}+\beta_{2}RPE+\varepsilon ,$$

where *PD_t_* indicates the trial-by-trial values of pupil dilations at time point *t* in the defined time window of 3 s. *ΔQ* indicates the trial-by-trial Q-value differences between the two paired-choice options in the choice phase. *Q_chosen_* and RPE denote the trial-by-trial value beliefs of the recently chosen options and reward predictor error in the feedback phase. The resulting regression coefficients in the choice and feedback phases for each participant were submitted to nonparametric cluster-based permutation *t* tests. It allowed us to examine the significant coefficients within or between age groups as well as correct multiple comparisons over time. The cluster size was determined by the number of continuous time series for which the *t* test resulted in *p <* 0.05, and then the observed cluster size was compared against a random cluster across subjects within or between age groups (1000 repetitions) to quantify the significance of this cluster in the time series. Note that the cluster-based permutation test was conducted using the MNE (version 0.20.7) package in Python, which not only examines the cluster-based difference between the distributions but also tests the timewise difference.

#### Age differences in LC-MRI contrast

LC-MRI contrast ratios were examined for age group differences in a 2 (age group) × 2 (hemisphere) × 3 (session) mixed ANOVA. The LC-MRI contrast ratios were averaged across hemispheres and sessions within participant for subsequent correlation analyses to acquire stabler intensity estimates.

#### Relationships among individual differences in value sensitivity, task-related pupil dilations, and LC integrity

To obtain a psychophysiological index of value uncertainty, the area under the curve of the pupil-related coefficient values across the whole time window (3 s; see Fig. 4*C–E*) in the choice or feedback phase was summed to represent the uncertainty-related effects on pupil dilations. The uncertainty (i.e., the associations with value beliefs) and RPE-evoked pupil dilations were further correlated with the β parameter values of the Q-learning model within each age group and across all participants after partialling out chronological age. We aimed to examine the relationship between individual and age differences in uncertainty or RPE-evoked pupil dilations and individual and age differences in value sensitivity during reward-based learning. Furthermore, we also correlated participants’ LC contrast ratios, which may be a potential indicator of NE functioning, with their β parameter values of the Q-learning model or with the uncertainty-evoked pupil dilations within each age group as well as across all participants. As not all data were normally distributed, Kendall’s rank correlation analyses were applied. Results from correlational analyses were Bonferroni corrected for multiple comparisons at an adjusted significance level of *p <* 0.05.

## Results

### Effects of age on choice performance and parameter estimates of the Q-learning model

**Figure 3.**
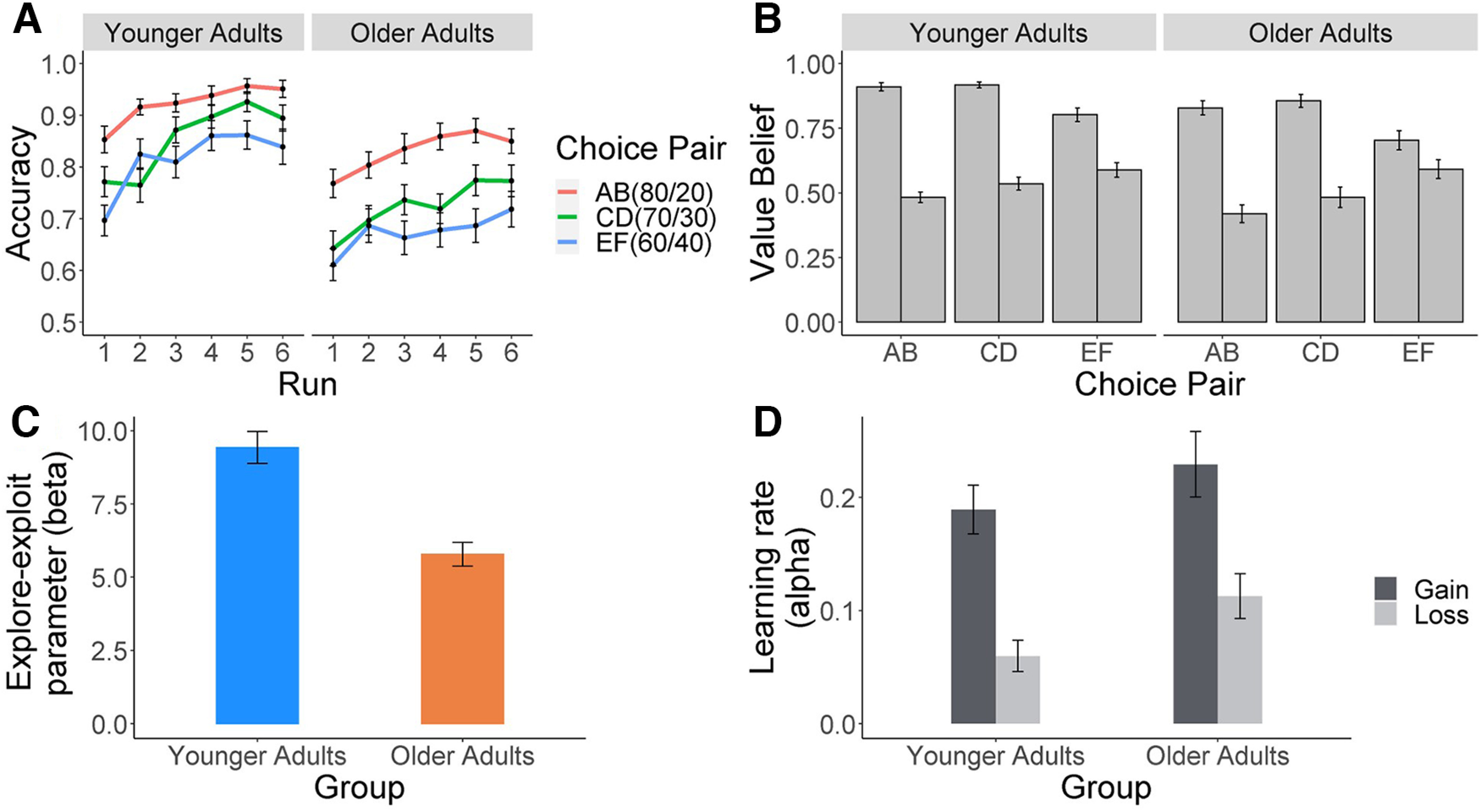
Choice performance and parameter estimates of the Q-learning model in both age groups. ***A***, Accuracy of selecting higher rewarded choice options. ***B***, Value belief estimates of the Q-learning model for each option at the end of the task. ***C***, Means of individual posterior distributions of the obtained estimates for β parameter. ***D***, Means of individual posterior distributions of the obtained parameter estimates for α_gain_ and α_loss_ parameters. Error bars indicate 1 SEM.

First, we examined age differences in the choice performance (Fig. 3*A*) for selecting higher rewarded options (i.e., the choice options with reward probabilities at 80, 70, and 60%). An aligned rank transform (nonparametric) ANOVA with age group as the between-subject factor (younger/older adults) and choice pair (80–20/70–30/60–40) and experimental run (1–6) as within-subject factors showed the main effects of age group (*F*_(1,93)_ = 24.71, *p <* 0.001, partial *η*^2^ = 0.21), choice pair (*F*_(2,1581)_ = 171.54, *p <* 0.001, partial *η*^2^ = 0.18), and experimental run (*F*_(5,1581)_ = 36.16, *p <* 0.001, partial *η*^2^ = 0.10) as well as significant interactions for group × choice pair (*F*_(2,1581)_ = 21.03, *p <* 0.001, partial *η*^2^ = 0.03) and for choice pair × experimental run (*F*_(10,1581)_ = 2.89, *p =* 0.001, partial *η*^2^ = 0.02). As anticipated, participants’ choice performance was best in the easiest choice pair with the highest difference of reward probability ratios (80/20) and decreased gradually as the difference of the probability ratios decreased (80/20–70/30, *t*_(1674)_ = 7.79, *p <* 0.001, *d =* 0.38; 70/30–60/40: *t*_(1674)_ = 3.93, *p <* 0.001, *d =* 0.19; Fig. 3*A*). In addition, both accuracies of both age groups increased in the last experimental run compared with the first run (run 1–6, *t*_(1674)_ = 7.12, *p <* 0.001, *d =* 0.35; Fig. 3*A*), indicating that participants were capable of learning about the probability knowledge from the feedback. Older adults exhibited worse choice performance than younger adults for all three choice pairs, and their accuracies decreased when the difference of reward probability ratios was reduced (80/20, *t*_(5.75)_ = 5.75, *p <* 0.001, *d =* 0.28; 70/30, *t*_(8.17)_ = 8.17, *p <* 0.001, *d =* 0.40; 60/40, *t*_(8.85)_ = 8.85, *p <* 0.001, *d =* 0.43).

**Figure 4.**
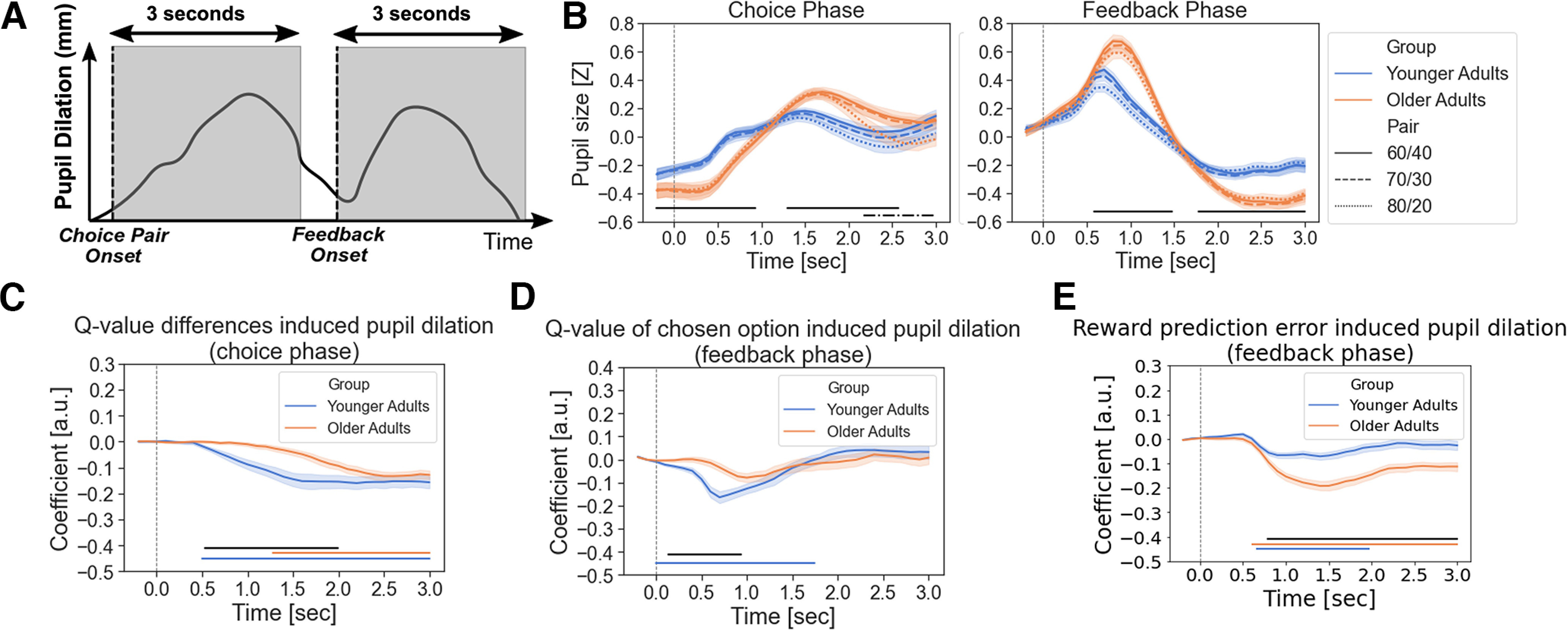
The associations among pupil dilations, value beliefs, and RPEs. ***A***, An example for depicting the pupillometry data analyzed within two separate time windows of interest covering 3 s after the choice pair or after the feedback. ***B***, Pupil dilations in each pair condition for both choice and feedback phases and both age groups. ***C***, Beta regression coefficients accounting for choice-related pupil dilations that were associated with the uncertainty of the reward probabilities between the two paired-choice options. After a choice pair was shown, smaller value differences between the choice options elicited larger pupil dilations in both age groups, but this effect was smaller and delayed in older adults. ***D–E***, Beta coefficients account for the feedback-related pupil responses that were associated with value updating of the recently chosen option. ***D***, Only younger adults’ pupil dilations negatively correlated with value beliefs of the recently chosen options. ***E***, In addition, both age groups showed negative correlations between pupil dilations and reward prediction errors. Lower-than-expected outcomes elicited larger pupil dilations, but this effect was stronger in older than younger adults. Note that (***B–E***) Lines and shaded areas represent the mean and SEM, respectively. Blue and orange horizontal lines at the bottom indicate the time points where regression coefficients significantly differentiate from zero in younger and older adults, respectively. Black horizontal lines indicate the time points showing significant age differences. Black dashed lines indicate the time points showing a significant main effect of the pair condition.

We also applied a Q-learning model with choice performance as the dependent variable to investigate value beliefs of the choice options at each trial and the individual’s exploitation-exploration tendency (also known as value sensitivity; see above, Materials and Methods for model description and analysis of parameter recovery). In particular, an aligned rank transform ANOVA with age group as the between-subject factor (younger/older adults) and the choice pair (80–20/70–30/60–40) as within-subject factors was applied to test for Q-value differences between the two choice options (Fig. 3*B*). Main effects of age group (*F*_(1,93)_ = 4.97, *p =* 0.02, partial *η*^2^ = 0.05) and choice pair (*F*_(1,186)_ = 103.30, *p <* 0.001, partial *η*^2^ = 0.55) as well as an interaction effect for age group × choice pair (*F*_(1,186)_ = 3.69, *p =* 0.03, partial *η*^2^ = 0.04) were observed. In both age groups, the value differences between the two choice options were largest for the 80/20 and 70/30, followed by the 60/40 choice pair (80/20–70/30, *t*_(279)_ = 1.74, *p =* 0.25, *d =* 0.21; 70/30–60/40, *t*_(279)_ = 9.17, *p <* 0.001, *d =* 1.10; Fig. 3*B*). Relative to younger adults, older adults showed lower differences in the value beliefs; however, this effect was mainly present in the choice pair with the smallest difference between the probability ratios (80/20, *t*_(279)_ = −0.54, *p =* 0.59, *d =* 0.06; 70/30, *t*_(279)_ = −0.28, *p =* 0.59, *d =* 0.03; 60/40, *t*_(279)_ = −3.06, *p =* 0.002, *d =* 0.36).

Furthermore, relative to younger adults, older adults exhibited a lower β parameter value (Mann–Whitney *U* = 1805, *p <* 0.001, *r =* 0.60; Fig. 3*C*), indicating lower value sensitivity and less exploitation with regard to the higher rewarded options. For the two learning rate parameters α_gain_ and α_loss_ (Fig. 3*D*), an aligned rank transform ANOVA with age group as the between-subject factor (younger/older adults) and learning type (gain/loss) as the within-subject factor (Fig. 3*D*) yielded a main effect on learning type (*F*_(1,93)_ = 81.61, *p <* 0.001, partial *η*^2^ = 0.47) but no main effect of age group (*F*_(1,93)_ = 1.25, *p =* 0.27, partial *η*^2^ = 0.01) or interaction for age group × learning rate (*F*_(1,93)_ < 1, *p =* 0.84, partial *η*^2^ < 0.01). Results showed that both age groups updated their value beliefs more from positive than negative feedback (Fig. 3*D*). In sum, in line with previous studies (Eppinger and Kray, 2011; Frank et al., 2007; Hämmerer et al., 2011), we observed that compared with younger adults, older adults showed worse choice performance in selecting higher rewarded options, less difference between the value beliefs in the most difficult choice pair (60/40), and smaller β parameter values of the Q-learning model, indicating lower value sensitivity to the reward probabilities.

### Effects of age on uncertainty-evoked pupil dilations during choice and feedback phases

To investigate age-related changes in value computation and updating during reward-based learning, we measured pupil size changes as a psychophysiological indicator and investigated its dependence on age and individual differences in value beliefs and RPEs during the task. To this end, we focused on two separate time windows covering 3 s after the choice pair or after the feedback was shown (Fig. 4*A*). The choice phase is associated with the value comparison between the two paired-choice options, whereas the feedback phase is related to the value estimation and updating of the recently chosen option. We found a main effect of age group (choice, all *p* values = 0.001, −0.2–0.93 s and 1.30–2.57 s postevent; feedback, all *p* values = 0.001, 0.59–1.47 s and 1.79–3 s postevent; Fig. 4*B*, solid black line) in both choice and feedback phases and a main effect of pair conditions in the choice phase (*p* = 0.001, 2.17–3 s postevent; Fig. 4*B*, black dashed line) on pupil dilations but no interactions (choice, all *p* values *>* 0.28; feedback, all *p* values *>* 0.17). Older adults overall exhibited larger pupillary responses compared with younger adults in both phases. Participants’ pupil dilations in the choice phase were largest for the 60/40 and 70/30, followed by the 80/20 choice pair, which might indicate a difficulty in the processing of value representations and comparisons.

Furthermore, our results showed that in the choice phase, younger adults’ pupil dilations negatively correlated with the Q-value differences between the two paired-choice options (*p_corr_* < 0.001, 0.5–3 s postevent; Fig. 4*C*, solid blue line). Lower value differences (i.e., greater decision uncertainty) elicited larger pupil dilations. Although older adults also exhibited this negative association (*p_corr_* = 0.001), the effect was smaller and only appeared later in time at 1.28–3 s relative to younger adults (Fig. 4*C*, solid orange line). Indeed, there was a significant age difference, indicating a smaller uncertainty effect in older than in younger adults (age difference, *p* = 0.006, 0.53–1.99 s postevent; Fig. 4*C*, solid black line). Although we found that older adults showed larger pupil responses than younger adults across all paired conditions (Fig. 4*B*, solid black line), the reduced uncertainty-evoked pupil dilations (i.e., associated with value beliefs) in the older age group may indicate noisy neural information processing of value representations and comparison (Li et al., 2001; Salthouse, 1996).

Furthermore, we found two component changes in pupil dilations after feedback onset. First, pupil dilations negatively correlated with value beliefs of the recently chosen options; however, this effect was only observed in younger (*p_corr_* = 0.001, 0–1.74 s postevent) but not in older adults (age difference, *p =* 0.02, 0.14–0.94 s postevent; Fig. 4*D*). The effect observed in younger adults may reflect the uncertainty about the value beliefs of the chosen options updated recently. The smaller uncertainty effect in the older age group might be associated with age-related impairments in value estimation and updating of the chosen option from the feedback. Second, compared with younger adults, older adults showed a larger negative association between RPEs and pupil dilations (younger, *p_corr_* = 0.004, 0.67–1.97 s postevent; older, *p_corr_* = 0.001, 0.61–3 s postevent; age difference: *p_corr_* = 0.001, 0.79–3 s postevent; Fig. 4*E*). In other words, lower-than-expected outcomes elicited larger pupil dilations. It might indicate that older adults still expected rewards in the choices associated with smaller value beliefs but received losses, thus, resulting in surprise because of negative RPE.

### Effects of age on the locus coeruleus contrast

To assess the LC-MRI contrast, the LC and pontine reference masks were applied to participants’ FLASH brainstem images that were moved to their whole-brain native space. The LC contrast ratio refers to the peak signal intensity in the LC masked region (Fig. 2*E*, red areas) relative to the peak signal intensity in the pontine reference masked region (Fig. 2*E*, blue areas) on each hemisphere and for each scan session. Table 3 depicts the mean and variability (SD) of signal intensities in the LC or pontine reference regions of interest across three scan sessions.

**Table 3:** Descriptive statistics of the signal intensities in the locus coeruleus and pontine reference regions of interest

ROI age group	LC		Pontine	
	Left hemisphere	Right hemisphere	Left hemisphere	Right hemisphere
Younger adults	720.97 (17.73)	695.14 (16.63)	661.68 (16.56)	649.66 (14.63)
Older adults	652.24 (19.61)	628.26 (20.24)	605.50 (15.94)	591.60 (18.49)

ROI, Region of interest. SDs are shown in parentheses.

**Figure 5.**
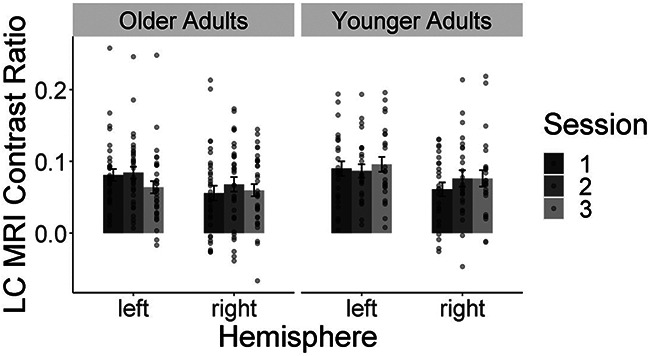
LC-MRI contrast ratio in both age groups. Bar plots depicting LC-MRI contrast ratios in each MRI scan session on the left or right hemisphere is displayed for younger and older adults. Younger adults overall showed a higher LC-MRI contrast ratio than older adults. The LC-MRI contrast ratios were higher on the left compared with the right hemisphere. Error bars indicate SEs, and scatter dots indicate the distribution of participants’ LC contrast ratios for each session and each hemisphere.

Specifically, when applying an aligned rank transform ANOVA on the LC-MRI contrast ratios with age group as the between-subject factor (younger/older adults) and hemisphere (left/right) and session (1/2/3) as within-subject factors, we found significant main effects of age group (*F*_(1,342)_ = 4.99, *p =* 0.03, *η*^2^ = 0.01) and hemisphere (*F*_(1,342)_ = 7.98, *p =* 0.005, *η*^2^ = 0.02) but no main effect of scan session or any interaction (all *F* values *<* 1, *p =* 0.48, *η*^2^ < 0.01). In general, the LC contrast ratio on the left hemisphere was higher than on the right hemisphere in both age groups (left, mean ± SD = 0.08 ± 0.05, range, −0.02–0.26; right, mean ± SD = 0.07 ± 0.06, range, −0.07–0.22; Fig. 5), as previously reported (Bachman et al., 2021; Betts et al., 2017). Relative to younger adults, older adults exhibited a lower LC contrast ratio (younger, mean ± SD = 0.08 ± 0.05, range, −0.05-0.22; older, mean ± SD = 0.07 ± 0.05, range, −0.06-0.22; Fig. 5), which might indicate the age-related degeneration of the LC structure (Jacobs et al., 2021a).

### Relationships among value sensitivity, uncertainty-evoked pupil dilations, and locus coeruleus contrast

**Figure 6.**
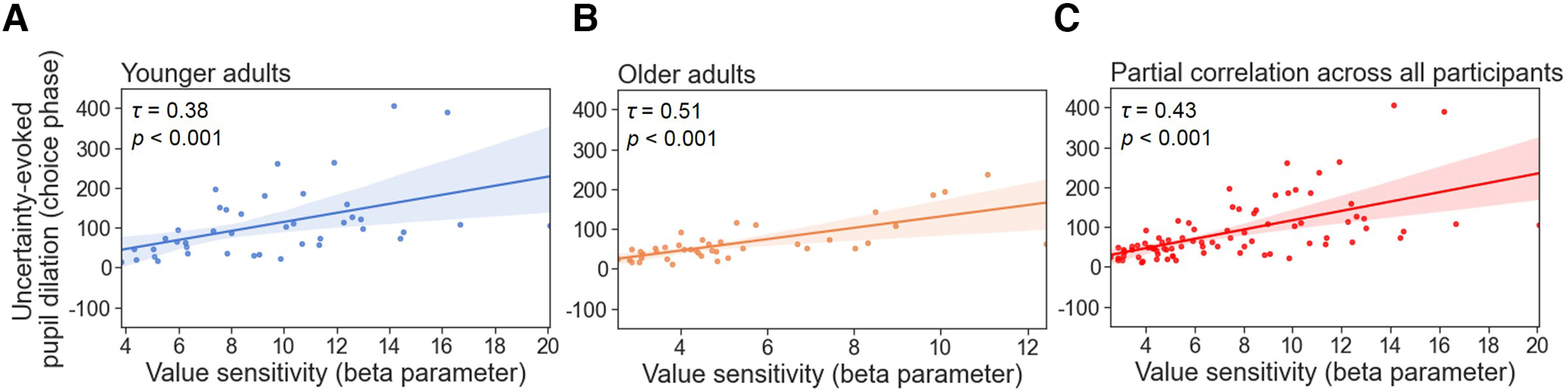
The associations between value sensitivities and uncertainty-evoked pupil dilations in the choice phase. ***A–C***, Scatter plots showing the correlations between the value sensitivities and the uncertainty-related effects on pupil dilations (beta coefficient) in younger (***A***) and older (***B***) age groups as well as across all participants after partialling out chronological age (***C***). Participants who showed a higher value sensitivity during reward-based learning exhibited a larger uncertainty-evoked pupil dilation in the choice phase. The shaded areas indicate the 95% confidence intervals. Correlation coefficients were computed using the Kendall rank correlation.

**Figure 7.**
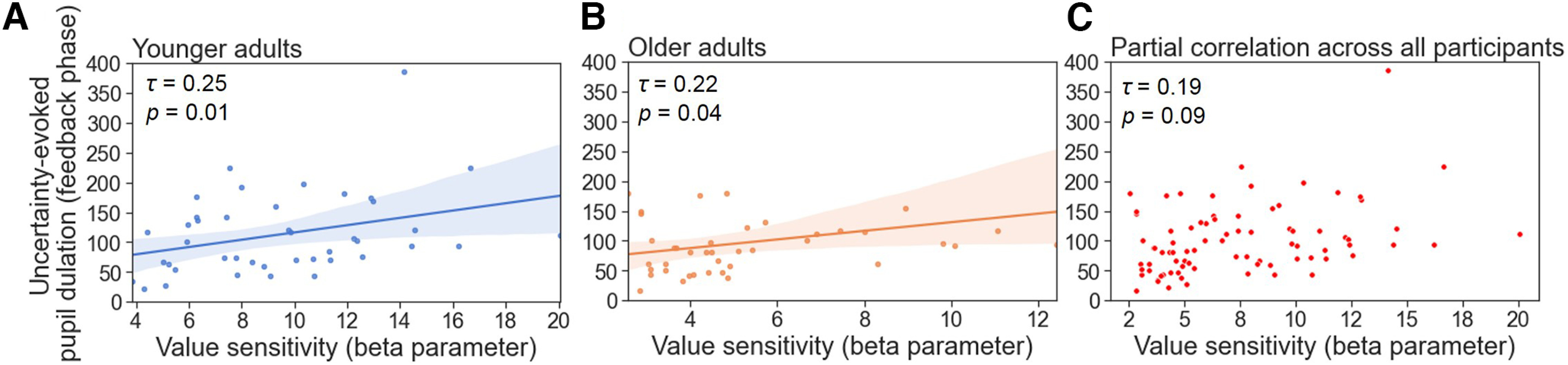
The associations between value sensitivities and uncertainty-evoked pupil dilations in the feedback phase. ***A–C***, Scatter plots showing the correlations between the value sensitivities and the uncertainty-related effects on pupil dilations (beta coefficient) in younger (***A***) and older (***B***) age groups as well as across all participants after partialling out chronological age (***C***). Both younger and older age groups showed a positive relationship. The shaded areas indicate 95% confidence intervals. Correlation coefficients were computed using the Kendall rank correlation.

To better understand the associations between the value sensitivity during reward-based learning and NE functioning, we correlated the individual’s β parameter values of the Q-learning model with (1) the uncertainty-related or RPE-related effects on an individual’s pupil dilations in the choice and feedback phases and with (2) the individual’s LC-MRI contrast. Because the main measure of interest in the pupillometry results was the extent of the uncertainty- or RPE-evoked pupil dilations, the area under the curve of the coefficient values across the time window (3 s) was summed up first. We used the summed pupil dilations to further correlate with the β parameter values within each age group as well as across all participants after partialling out the chronological age. In the choice phase, β parameter values positively correlated with the uncertainty-evoked pupil dilations in each age group (younger, Kendall’s *τ* = 0.38, *p <* 0.001; Fig. 6*A*; older, Kendall’s *τ* = 0.51, *p <* 0.001; Fig. 6*B*) and across all participants after partialling out the chronological age (Kendall’s *τ* = 0.43, *p <* 0.001, Fig. 6*C*). Individuals who showed a higher value sensitivity exhibited larger uncertainty about the choice options given small Q-value differences. We also found similar patterns of associations in the feedback phase. The β parameter values were positively correlated with the uncertainty-evoked pupil dilations in both age groups (younger, Kendall’s *τ* = 0.25, *p =* 0.01; Fig. 7*A*; older, Kendall’s *τ* = 0.22, *p =* 0.04; Fig. 7*B*) and a trendwise association across all participants after partialling out the chronological age (Kendall’s *τ* = 0.19, *p =* 0.09; Fig. 7*C*). Participants who showed a higher value sensitivity exhibited stronger uncertainty-evoked pupil dilations associated with small value beliefs of the chosen options in the feedback phase, which might lead to better value estimation and updating from feedback learning. In addition, we found no associations between β parameter values and RPE-evoked pupil dilations in younger (Kendall’s *τ* = 0.05, *p =* 0.63) or older adults (Kendall’s *τ* = 0.09, *p =* 0.42) or across all participants after partialling out the chronological age (Kendall’s *τ* = 0.04, *p =* 0.73). These findings might indicate individual differences in value computation and updating during decision-making and reward-based learning.

**Figure 8.**
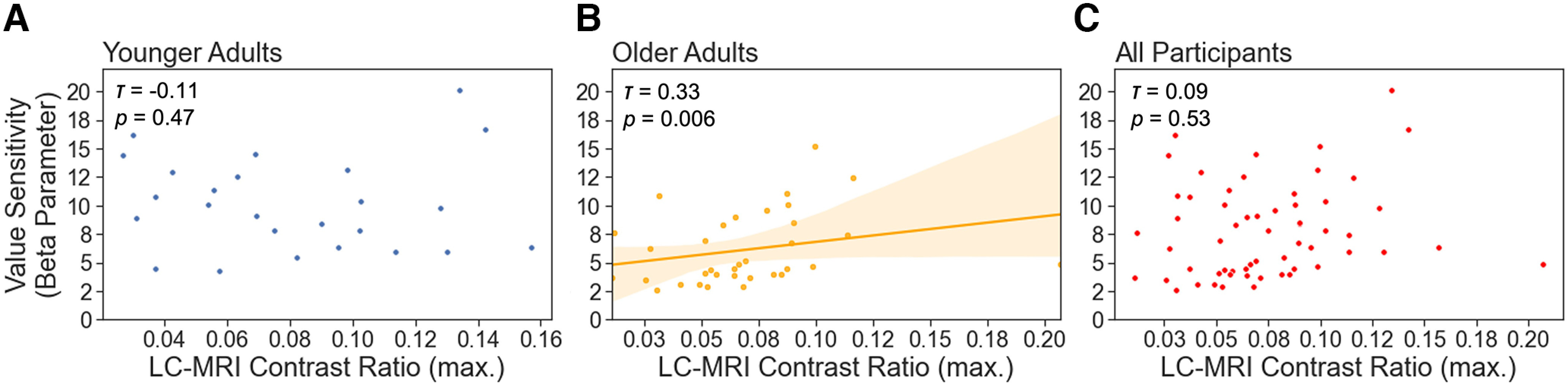
The associations between value sensitivities and LC-MRI contrast ratios. ***A–C***, Scatter plots showing the correlations between the value sensitivities and the LC-MRI contrast ratios in younger (***A***) and older (***B***) age groups as well as across all participants after partialling out chronological age (***C***). Only older adults who showed a higher LC-MRI contrast ratio exhibited a higher value sensitivity during reward-based learning. The shaded areas indicate 95% confidence intervals. Correlation coefficients were computed using the Kendall rank correlation.

For the LC-MRI contrast, there was no significant correlation between the β parameter values and the LC-MRI contrasts in younger adults (Kendall’s *τ* = −0.11, *p =* 0.47; Fig. 8*A*) or across all participants (Kendall’s *τ* = 0.09, *p =* 0.53; Fig. 8*C*), but there was a positive association in older adults (Kendall’s *τ* = 0.33, *p =* 0.006; Fig. 8*B*). Using the Fischer *r*-to-*z* transformation to test the difference between these correlations, we found a significant difference in the observed results between the two age groups (*z =* 2.49, *p =* 0.006). In addition, we also explored whether LC-MRI contrast would relate to uncertainty-related effects on pupil dilations. Results showed no significant correlation between the LC contrast ratios and the uncertainty-evoked pupil dilations in the choice (younger, Kendall’s *τ* = −0.03, *p =* 0.86; older, Kendall’s *τ* = 0.18, *p =* 0.20; across all participants, *r =* 0.07, *p =* 0.65) or feedback phase (younger, Kendall’s *τ* = 0.27, *p =* 0.07; older, Kendall’s *τ* = 0.13, *p =* 0.34; across all participants, Kendall’s *τ* = 0.20, *p =* 0.16) or between the LC contrast ratios and RPE-induced pupil dilations in the feedback phase (younger, Kendall’s *τ* = −0.15, *p =* 0.34; older, Kendall’s *τ* = 0.12, *p =* 0.39; across all participants, Kendall’s *τ* = −0.01, *p =* 0.95). In sum, we found a positive correlation between participants’ uncertainty-induced pupil dilations in both choice and feedback phases and the value sensitivities as reflected in the β parameter values of the Q-learning model. Only older adults’ LC-MRI contrast ratios were positively associated with their value sensitives.

## Discussion

To investigate how the age-related decline in NE functioning may affect decision-making and reward-based learning, we conducted a probabilistic decision-making task and applied a reinforcement Q-learning model to capture individuals’ learning tendency characteristics in younger and older adults. Moreover, pupillary responses during the task and structural LC-MRI contrast were measured in both age groups to serve as windows of NE functioning. The present results not only showed reduced value sensitivity to reward probabilities in the older compared with the younger age group but also indicated the contribution of the LC-NE system to reward-based learning in old age.

Relative to younger adults, older adults in the present study showed lower accuracy in selecting higher rewarded options and lower β parameter values of the Q-learning model, indicating reduced value sensitivity to reward probabilities. These findings of adult age differences are in line with previous studies that have shown deficits in choice performance during reward-based learning in old age (Eppinger and Kray, 2011; Frank et al., 2007; Hämmerer et al., 2011). The reduced value sensitivities in older adults may be related to age-related decline in the DA and/or NE functioning underlying the processes of decision-making and reward-based learning in the frontostriatal network (Chowdhury et al., 2013; de Boer et al., 2017; Dreher et al., 2008; Sales et al., 2018).

Empirical evidence has shown that pupil size fluctuations vary as a function of states related to reward-based learning (Hämmerer et al., 2019; Manohar and Husain, 2015; Muhammed et al., 2016; Van Slooten et al., 2018). In our study, younger adults’ pupil dilations were negatively related to the trial-by-trial basis of value beliefs in both choice and feedback phases. In the choice phase, smaller value differences between the paired-choice options elicited larger pupil dilations. This effect may reflect the uncertainty to the value comparison between the two paired-choice options. In the feedback phase, we found that smaller value beliefs of the chosen options elicited larger pupil dilations, indicating the uncertainty about the value estimation and updating of the recently chosen options from the feedback. These findings are in line with the results of previous studies demonstrating that pupils dilated when value uncertainty increased (Lavín et al., 2014; Urai et al., 2017; Van Slooten et al., 2018). Contrary to younger adults, the uncertainty effects on pupil dilations were smaller and appeared later in older adults in the choice phase, and they were also smaller in the feedback phase. The temporal delay and smaller effects of uncertainty during decision-making in the older age group may indicate noisy neural information processing of the value representations and comparison (Li et al., 2001; Salthouse, 1996). After feedback, the smaller uncertainty effects observed in older adults may reflect suboptimal value estimation and updating, resulting in their decision tendency less toward exploitations to the higher rewarded options and irrational reward expectations to the lower rewarded options. This suggestion could be underpinned by the latter effect in the feedback phase in which older adults showed a larger negative correlation between pupil dilations and RPEs. Lower-than-expected outcomes elicited larger pupil dilations, indicating a surprise because of negative RPE (Lavín et al., 2014; Preuschoff et al., 2011; Van Slooten et al., 2018). Altogether, pupil dilations during the probabilistic decision-making task not only scaled with value uncertainty but also revealed an age-related decline in value estimation and updating during reward-based learning.

Furthermore, we tested the degree to which individual differences in value sensitivity (β parameter values) during reward-based learning may be related to individual differences in uncertainty-evoked pupil dilations. In both choice and feedback phases, results showed that participants’ β parameter values of the Q-learning model positively correlated with the uncertainty-evoked pupil dilations (Figs. 6, 7). In other words, individuals who had a higher value sensitivity to the reward probabilities showed larger uncertainty-evoked pupil dilations. Our findings are in line with previous literature, which demonstrated that individuals who had optimal performance in selecting higher rewarded options exhibited increased task-related pupil dilations during probabilistic decision-making and reward-based learnings (Silvetti et al., 2013; Van Slooten et al., 2018).

*In vivo* pupillometry has been demonstrated as a noninvasive readout of LC activity (Breton-Provencher and Sur, 2019; Joshi et al., 2016; Privitera et al., 2020). Although a previous study (Megemont et al., 2022) showed that pupillary responses are not directly associated with the tonic firing rates of LC neurons, task-related pupil dilations have been associated with phasic activity within the LC neurons in animal (Joshi et al., 2016; Reimer et al., 2016; Usher et al., 1999; also see Megemont et al., 2022 for discussion) and in human studies (de Gee et al., 2014; Murphy et al., 2014). In the present study, one could speculate that our pupillometry findings might indicate age and individual differences in the phasic LC function associated with value representations and updating during reward-based learning. However, we did not directly assess the phasic activation of the LC. It is difficult to conclude the relationship between pupil dilations and age-related changes in the phasic activity of the LC. In addition, pupil dilation is also affected by other neurotransmitters such as serotonin and acetylcholine (Cazettes et al., 2021; Wang et al., 2017; Yu et al., 2004). Future studies combined with pupillometry and a neuroimaging approach (e.g., fMRI) are needed to clarify the associations between uncertainty-evoked pupil dilation and phasic LC activity during decision-making and reward-based learning in aging.

Finally, we found adult age differences in the LC-MRI contrast. Relative to younger adults, the LC contrast was lower in older adults. Thus far, evidence on the age differences of the LC-MRI contrast showed mixed findings. Some studies reported a higher LC contrast ratio in older than in younger adults (Betts et al., 2017; Clewett et al., 2016), but others showed no difference between age groups (Gallant et al., 2022; Hämmerer et al., 2018). Previous studies that recruited participants from 20 to 80 years of age reported a quadratic relationship of the LC-MRI contrast across the lifespan (Jacobs et al., 2021b; Liu et al., 2017; Shibata et al., 2006). Their results further illustrated that the LC-MRI contrast showed a peak at ∼60 years and then gradually decreased in late adulthood age. The heterogeneous findings about age differences in the LC-MRI contrast might be because of the comparisons between different cross-sectional age groups. Despite these mixed findings in adult age differences of the LC-MRI, previous studies showed that the LC-MRI contrast positively correlated with memory performance (Dahl et al., 2019; Hämmerer et al., 2018) and with cortical thickness (Bachman et al., 2021) only in older adults. In the present study, we also found an association between the LC-MRI contrast and value sensitivity (β parameter) during reward-based learning in older but not in younger adults. It suggests that losses in anatomic (e.g., the LC structure) brain resources in normal aging may render a stronger coupling of cognitive heterogeneity among individual variations in the neurocognitive efficacy (Lindenberger et al., 2008). Although we did not find any association between the LC-MRI contrasts and task-related pupil dilations, this lack of association could be because of the small sample size (only 25 younger and 34 older adults). Previous studies (Berger et al., 2023; Gallant et al., 2022; Hämmerer et al., 2018) with a similar sample size (∼30 younger and ∼30 older adults) also reported no associations between arousal-evoked pupil dilations and the LC-MRI contrast. We know little about the relationship among the LC structure, its phasic activity, and its neurochemical function in modulating cognition. Some studies using positron emission tomography tracers such as [^18^F]Fluoro-m-tyrosine demonstrated the availability of measuring LC norepinephrine synthesis capacity (Ciampa et al., 2022; Ono et al., 2016). Future studies using functional and neuromodulator imaging may comprehensively clarify the associations between age-related changes in reward-based learning and the LC-NE system.

In conclusion, our study demonstrated that task-related pupil dilations can track age-related deficits in value estimation and updating during reward-based learning. The findings further indicated the association between uncertainty-evoked pupil dilations and value sensitives, as quantified by the β parameter of our Q-learning model. Moreover, we found adult age differences in the LC-MRI contrast and a positive association between the LC-MRI contrast and value sensitivity during the task only in older adults. These findings may indicate age-related declines of LC functioning in modulating value computation and updating during decision-making and reward-based learning.
